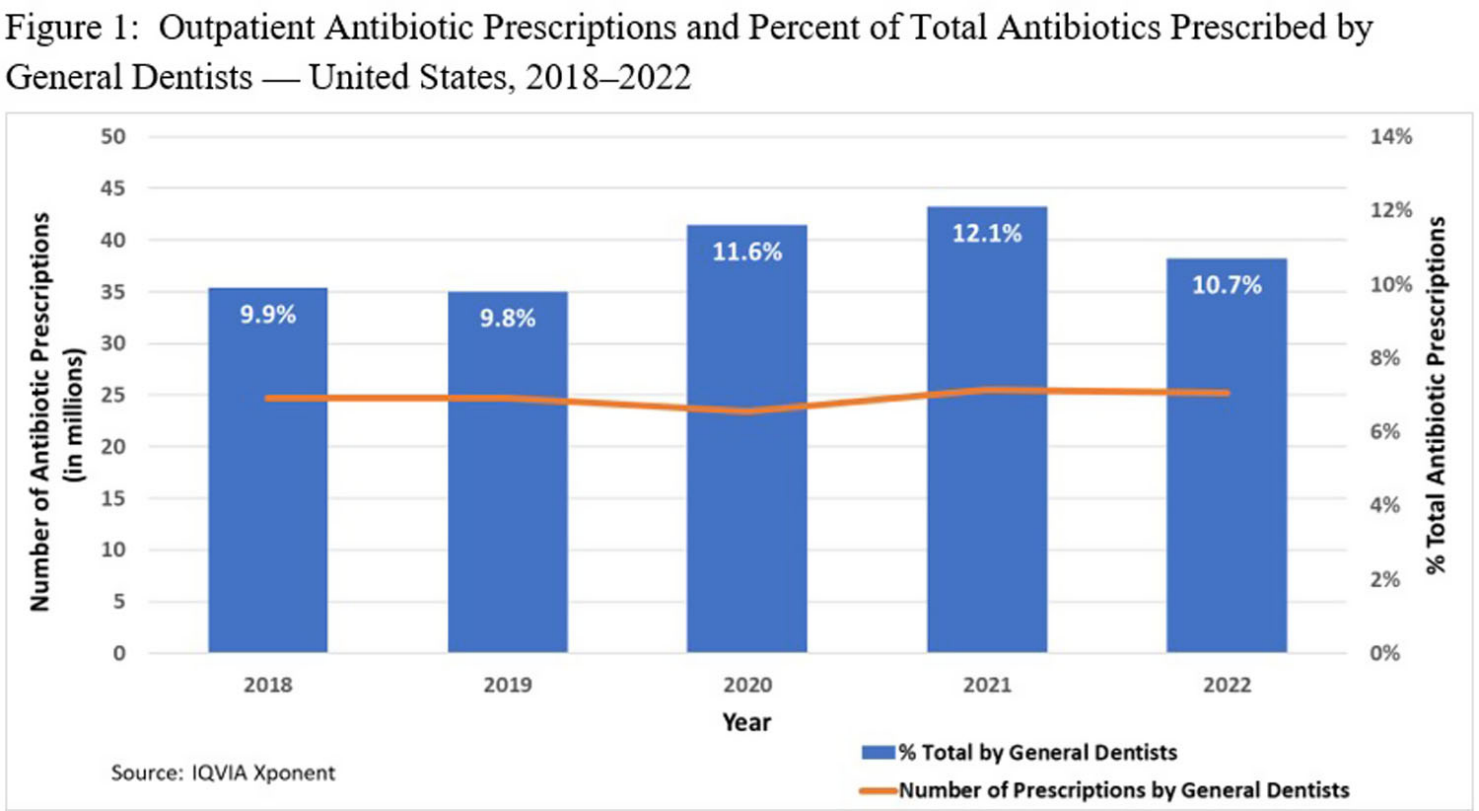# Antibiotic Prescribing by General Dentists in the Outpatient Setting — United States, 2018–2022

**DOI:** 10.1017/ash.2024.125

**Published:** 2024-09-16

**Authors:** Cam-Van Huynh, Katryna Gouin, Sarah Kabbani, Lauri Hicks, Michele Neuburger, Emily McDonald

**Affiliations:** Centers for Disease Control and Prevention; DHQP

## Abstract

**Background:** Inappropriate antibiotic use impacts patient safety and antimicrobial resistance patterns. In 2013, general dentists in the U.S. prescribed nearly 10% of all outpatient oral antibiotics (24.5 million prescriptions). The American Dental Association (ADA) published guidelines in 2019 recommending limited antibiotic prescribing for the treatment of dental pain and swelling. We characterized dental prescribing during 2018–2022 to assess whether antibiotic use decreased after the guideline’s release. In addition, we examined access to dental care. **Methods:** All antibiotic prescriptions dispensed during 2018–2022 were extracted from the IQVIA Xponent database, which captured ≥92% of all U.S. outpatient prescriptions and projected to 100% coverage. Prescriptions by general dentists were compared to total outpatient oral antibiotic prescriptions and summarized by patient sex, patient age, and prescriber geographic region. Census denominators were used to calculate prescribing rates per 1,000 persons. IQVIA general dentist counts were used to calculate dentists per 100,000 persons. **Results:** General dentists prescribed 24.7 million antibiotic prescriptions in 2018 (75 prescriptions per 1,000 persons) compared with 25.2 million (76 prescriptions per 1,000 persons) in 2022. During 2020–2022, general dentists prescribed >10% of all outpatient antibiotic prescriptions (range 10.7%–12.1%). In each year, prescription rates were higher for females, patients > 65 years, and among prescribers in the Northeast. In 2022, there were 58 general dentists per 100,000 persons in the United States. The highest general dentist rate was in District of Columbia (100 per 100,000 persons) and the lowest rate was in Delaware (41 per 100,000 persons). **Conclusions:** Despite the ADA’s 2019 guidelines, prescribing by general dentists remained stable during 2018–2022. Because the total number of antibiotic prescriptions overall decreased, general dentists’ share of all outpatient antibiotic prescriptions increased to >10% in recent years. Rate variation by patient characteristics and prescriber region may reflect differences in dental disease burden or may represent unnecessary antibiotic use. Dental antibiotic stewardship is needed, including dissemination and implementation of current prescribing guidelines. Further evaluation of prescribing indications and access to dental care is needed to inform dental stewardship priorities.

**Figure:**